# Presumptive *Dipylidium caninum* Infection in a Toddler

**DOI:** 10.1155/2020/4716124

**Published:** 2020-03-07

**Authors:** Hannah F Chong, Roukaya Al Hammoud, Michael L Chang

**Affiliations:** ^1^Memorial Hermann Hospital, Houston, TX, USA; ^2^The University of Texas Health Science Center, McGovern Medical School, Pediatric Infectious Diseases Division, 6431 Fannin, MSB 3.126, Houston, TX 77030, USA

## Abstract

We report a female toddler who presented repetitively with a chief complaint of motile white worms seen in her diapers. Symptoms of perianal itching and visualization of visible motile worms persisted for 6 months despite being treated with multiple courses of albendazole causing a lot of frustration and distress to the caregivers. The characteristics of the worms by inspection along with the presence of 3 pet dogs are consistent with *Dipylidium caninum*.

## 1. Introduction

A previously healthy 2-year-old girl presented with numerous small, white, visibly moving worms in her stool daily for 6 months. She had perianal pruritus but no fever, diarrhea, vomiting, or abnormal weight gain. Her pediatrician prescribed 2 doses of albendazole for suspected diagnosis of pinworms. Stool specimens were sent on six separate occasions for microscopic examination for ova and parasites and were reported as negative each time. Fecal occult blood testing and stool cultures for bacterial pathogens were also negative. All family members were prescribed albendazole, under the presumption of recurring pinworm infection. Despite negative testing and multiple courses of albendazole, the mother continued to see live worms, and the patient developed increased irritability and night-time fussiness. The mother became frustrated with her pediatrician. She then was referred to a pediatric infectious diseases specialist.

The patient lived with both parents in a rural area near Houston, Texas, and had no siblings. The patient did attend daycare. The family had no history of travel outside the Houston area. No other family members had similar symptoms. The family denied eating undercooked meat or seafood. They had three dogs which had access to outdoors and one cat inside the house. No history of fleas on pets or in the house or regular treatment of the pets for fleas.

Physical examination was normal except for mild dermatitis in the perineal area with linear excoriations thought to be secondary to the patient scratching. No obvious worms were visualized. The mother presented video footage and pictures of the worms in the stool from her smartphone. The video revealed motile, white worms about 1 centimeter in length (see Supplementary Materials). Based on the history and visual footage, the child was presumptively treated with one dose of praziquantel (10 mg/kg) for *Dipylidium caninum* infection followed by complete resolution of her symptoms.

## 2. Discussion

*Dipylidium caninum*, also known as flea tapeworm, is the most common cestode (tapeworm) of dogs and cats in the United States. Infection rates range from 0.1 to 4.0% for dogs in the North America [1]. One study identified *Dipylidium caninum* in 49.5% of nontreated dogs in animal shelters in Northern Oklahoma [1]. Using a new PCR method that identifies *Dipylidium caninum* rDNA inside single fleas in pet dogs and cats, out of 1969 *Ctenocephalides felis* from cats, 2.23% were found to be infected with *Dipylidium* and from 396 dogs infested with *Ctenocephalides canis*, 9.1% were infested with the *Dipylidium*-infected fleas [2]. The life cycle of this cestode starts with gravid proglottids that are passed in the feces or emerges from the perianal region of the host. The proglottids release egg packets which are consumed by flea larvae, the intermediate host. Eggs mature into infective cysticercoid larvae within the flea larvae as they mature to adults. Adult fleas containing cysticercoids are then ingested by dogs, the principal definitive hosts. Humans are usually infected via accidental ingestion of the intermediate host, cysticercoid-infected fleas. In the small intestine of the host (humans or dogs/cats), the cysticercoid develops into an adult tapeworm that produces proglottids which are later released into feces completing the life cycle [3]. Proglottids of the *Dipylidium caninum* are usually 12 mm in length and similar in appearance to rice grains.

Human infections, mostly in infants and children, occur worldwide but are uncommonly reported, likely representing under-reporting. In 2008, Samkari et al. reported one pediatric case and reviewed cases reported in the English-language literature which revealed a total of 34 cases since 1950s [4]. Since that publication, a total of 9 individual pediatric cases have been reported in journals indexed by PubMed, though only 4 are in English language [5–13]. An additional case series of 10 pediatric patients were reported in Greece [14]. Most *Dipylidium* infections are asymptomatic and self-limited, though abdominal pain, restlessness, and agitation can occur. The most notable sign is the presence of proglottids in the stool, diapers, or on the perineum. The duration of symptoms in our patient appears unusual, and it is possible that she had multiple reinoculations.

The gold standard diagnosis of *Dipylidium caninum* is made by visualizing the distinctive characteristics of proglottids which are usually highly motile when freshly passed. Unlike proglottids of *Taenia* species which are more square in shape, they are longer than they are wide (average mature size 12 mm × 3 mm), appears as rice grains or cucumber seeds, and have two sets of male and female reproductive organs. They therefore demonstrate two genital pores that lead to their name as the “double-pored” tapeworm. Proglottids of different *Taenia* species can be identified by their uterine branches. Within the gravid proglottids of *Dipylidium caninum* are egg packets. These eggs are round to ovoid, and their numbers can range from 5 to 30. Stool examination for ova is typically negative as eggs are rarely released from the proglottids within humans. Patient did not have risk factors (eating undercooked meat or fish) for other tapeworms that may infect humans as *Taenia saginata*, *Taenia solium*, *Diphyllobothrium latum* or others.

Affected children are often treated multiple times for *Enterobius* infection with albendazole, which is ineffective against *Dipylidium*. Families and primary care providers who are unfamiliar with the infection can become frustrated when the treatment seems to fail repeatedly which occurred in our case. The treatment of choice in the United States is a single dose of praziquantel (5 to 10 mg/kg). Praziquantel resistance in unresolving *Dipylidium* infection in dogs has been recently reported. These dogs were later successfully treated with nitroscanate or a compounded pyrantel/praziquantel/oxantel product [15]. Importantly, prevention of human cases should include primarily prompt parasitic treatment of the pets and flea control in their indoor and outdoor environments along with appropriate disposal of their feces, discouraging children to play in areas soiled with pets' feces, and good hand hygiene after playing with animals or outdoors.

In conclusion, we present a 2 year-old infected with *Dipylidium caninum* presumptively diagnosed by visualizing the characteristic motile proglottids from the video provided by the mother. We bring attention to this case in order to facilitate diagnosis and minimize provider and parent frustration when persistent pinworm infection is being considered.

## Supplementary Materials

Supplementary Materials The video revealed motile, white worms about 1 centimeter in length.
